# A Rare Case of Atypical Anti-glomerular Basement Membrane Disease

**DOI:** 10.7759/cureus.49064

**Published:** 2023-11-19

**Authors:** Sushrut Gupta, Pranjal Kashiv, Kapil N Sejpal, Shubham Dubey, Sunny Malde, Prasad Gurjar, Vrushali Mahajan, Amit Pasari, Manish Balwani

**Affiliations:** 1 Nephrology, Jawaharlal Nehru Medical College, Datta Meghe Institute of Higher Education and Research, Wardha, IND; 2 Pathology, Alexis Multispeciality Hospital, Nagpur, IND; 3 Nephrology, Saraswati Kidney Care Centre, Nagpur, IND

**Keywords:** percutaneous kidney biopsy, rapidly progressive renal failure, therapeutic plasmapheresis, atypical anti-glomerular basement disease, anti-gbm disease

## Abstract

Anti-glomerular basement membrane (Anti-GBM) disease is a severe form of glomerulonephritis (GN) that predominantly impacts individuals aged 20 to 70. It arises from the presence of circulating antibodies that specifically target an antigen inherent to the basement membranes of glomerular and alveolar structures. A unique subset within this category is termed atypical anti-GBM disease. In this variant, a distinctive feature is the widespread linear staining of the glomerular basement membrane (GBM) by IgG observed through immunofluorescence microscopy, with the notable absence of anti-GBM antibodies in the patient's serum. Here, we present an unusual case involving a 65-year-old female patient who sought medical attention due to rapidly progressing renal failure. The initial management included six hemodialysis sessions. Following a kidney biopsy, the diagnosis revealed a sclerosed phase of diffuse crescentic glomerulonephritis, attributed to atypical anti-GBM disease. Given the presence of diffuse crescents on the kidney biopsy, the medical team opted for an aggressive treatment regimen, commencing with intravenous methylprednisolone, followed by oral cyclophosphamide and oral prednisolone. Plasmapheresis was also recommended as part of the treatment plan, although it did not materialize due to the family's reluctance. Despite exhaustive efforts, the renal failure exhibited no signs of improvement, leading to the patient's discharge with a plan for ongoing maintenance hemodialysis. It is crucial to emphasize the pivotal role of immunosuppressive medications in managing this condition, as they play a critical role in preventing antibody formation and subsequent hypersynthesis that can exacerbate the disease.

## Introduction

Anti-glomerular basement membrane (anti-GBM) disease, a variant of small vessel vasculitis, involves circulating antibodies that target an antigen intrinsic to both glomerular and alveolar basement membranes [1]. Classified as an autoimmune disorder, it contributes to around 20% of rapidly progressive renal failure cases. With an incidence rate as low as one in 1,000,000, it stands out as one of the most severe forms of acute glomerulonephritis (GN) [1]. Its typical presentation includes swiftly advancing renal failure, with or without lung complications. In 60-80% of instances, patients face kidney and lung issues, while the rest may encounter kidney-specific or, in rare cases, pulmonary-only symptoms [2].

Although anti-glomerular basement membrane disease can affect individuals of any age, it peaks in incidence during the third and sixth decades of life. The five-year renal and patient survival rates are 34% and 83%, respectively. The condition primarily stems from circulating autoantibodies targeting epitopes in the noncollagenous 1 domain of type IV collagen's alpha-3 chain (a3NC1) [3]. Most patients show detectable serum anti-GBM antibodies, yet there's a variant known as atypical anti-GBM disease. IgG antibodies result in widespread linear staining of the glomerular basement membrane in this variant under immunofluorescence microscopy. However, enzyme-linked immunosorbent assay (ELISA), western blot, or indirect immunofluorescence assays fail to detect circulating serum anti-GBM antibodies [4]. Despite being previously considered less aggressive, atypical anti-GBM disease can still lead to end-stage renal disease (ESRD) in 15-32% of cases within a relatively short timeframe.

We present a case of atypical anti-GBM disease characterized by diffuse crescentic membranoproliferative glomerulonephritis. The first recorded instance of atypical anti-GBM disease dates back to 1993 [3]. The patient displayed proteinuria and hematuria but maintained normal kidney function. The kidney biopsy revealed focal crescents involving less than 50% of the glomeruli, with no membranoproliferative features. In contrast, our patient experienced rapidly progressive glomerulonephritis (RPGN) and received a diagnosis of diffuse crescentic membranoproliferative GN, affecting over 50% of the glomeruli. Approximately 2% to 8% of anti-GBM disease patients may exhibit negative serology results despite thorough testing [5]. In 2016, Nasr et al. [5] documented the most extensive case series, featuring 20 patients with atypical anti-GBM disease.

## Case presentation

A 65-year-old woman, previously without symptoms, experienced a notable decline in health approximately one month ago, presenting with issues like lower limb swelling, decreased urine output, and shortness of breath. Significantly, she had no previous history of renal dysfunction or any underlying medical conditions. Upon admission, an extensive array of investigations was carried out, and the findings are outlined in Table 1. It is crucial to highlight that all viral and immunologic assessments produced negative results. Considering the possibility of rapidly progressive renal failure (RPRF), a kidney biopsy was undertaken.

**Table 1:** Laboratory parameters done on admission HPF: High Power Field; ELISA: Enzyme-Linked Immunosorbent Assay

Lab Parameters	Values	Normal Values
Haemoglobin	7.2 g/dL	12-15 grams per deciliter (gm/dL)
White Blood Cells	9500 cells/μL	4000-10000 cells per microliter (cells/μL)
Platelets	2.72 l/L	150,000-450,000/μL
Blood Urea	120 mg/dL	15-36 milligrams per deciliter (mg/dL)
Creatinine	7.5 mg/dL	0.5-1.04 mg/dL
Albumin	2.6 g/dL	3.5-5 grams per deciliter (g/dL)
Urine Albumin	2+	
Urine Pus Cells	4-5 cells/HPF	
Red Blood Cells	Abundant	
Anti-GBM Antibodies (Method - Indirect Immunofluorescence)	Negative	
Antinuclear Antibodies Profile (Method - Immunoblot (Western Blot))	Negative	
Anti-Neutrophil Cytoplasmic Antibodies (Method - ELISA)	Negative	
Complement 3 Levels (Method - Immunoturbidimetry)	90-180 mg/dL	90-180 milligrams per deciliter (mg/dL)
Complement 4 Levels (Method - Immunoturbidimetry)	10-40 mg/dL	10-40 milligrams per deciliter (mg/dL)

The microscopic analysis of kidney tissue indicated the presence of suboptimal sampling. Four demonstrated global sclerosis within these glomeruli, while five showed crescent formation. Among these, three crescents were primarily fibrous, and the remaining two were fibrocellular. One glomerulus did not exhibit any crescent formation. Additionally, mild acute tubular injury was noted. Direct immunofluorescence studies identified three glomeruli with intense linear staining of the glomerular basement membrane (GBM) for IgG (3+), lambda (1+), and kappa (1+). Other findings included mild to moderate interstitial fibrosis and tubular atrophy (IFTA), affecting around 35% of the kidney tissue and changes related to the vessels.

**Figure 1:**
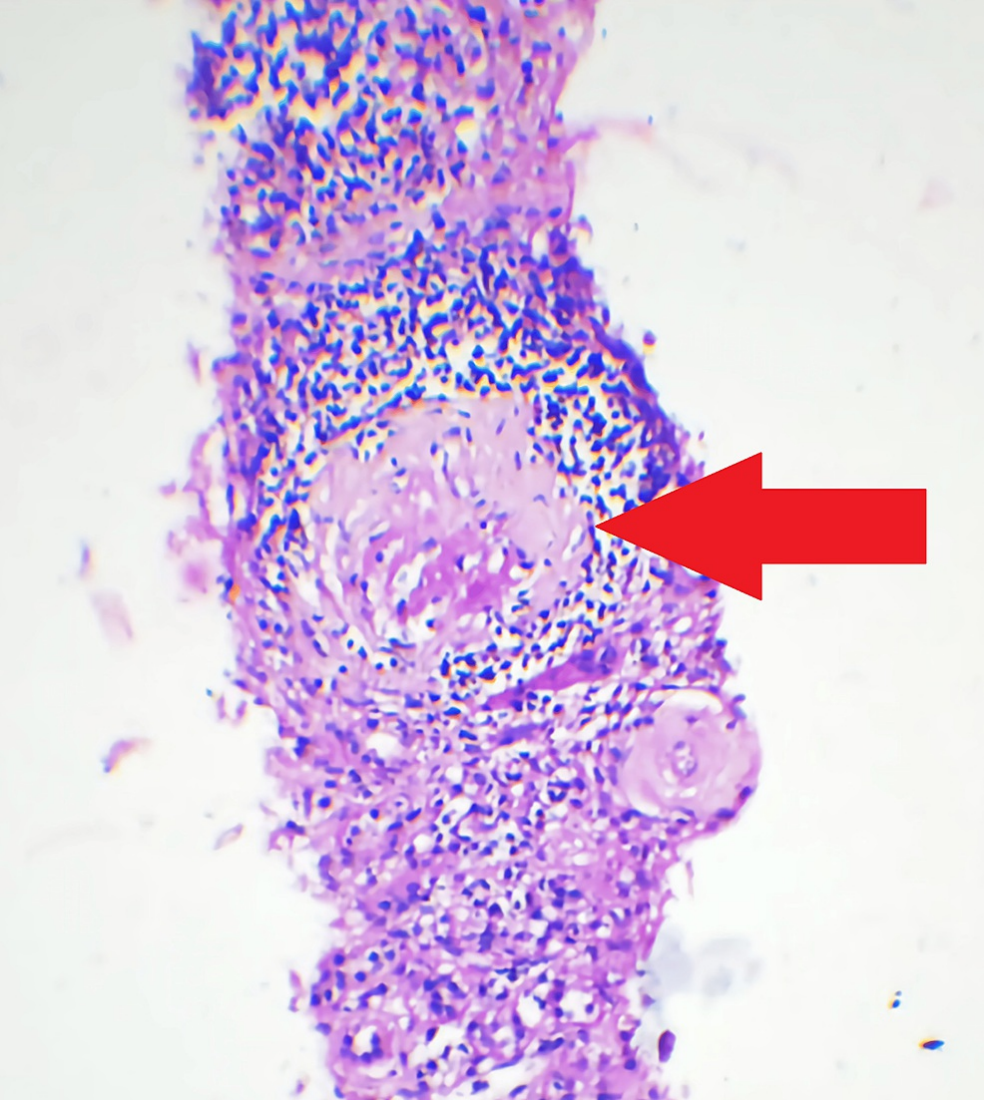
Periodic-acid-Schiff (PAS) stain 10X microphotograph showing mostly sclerosed glomeruli. One glomerulus with a fibrous crescent is seen (red arrow)

**Figure 2:**
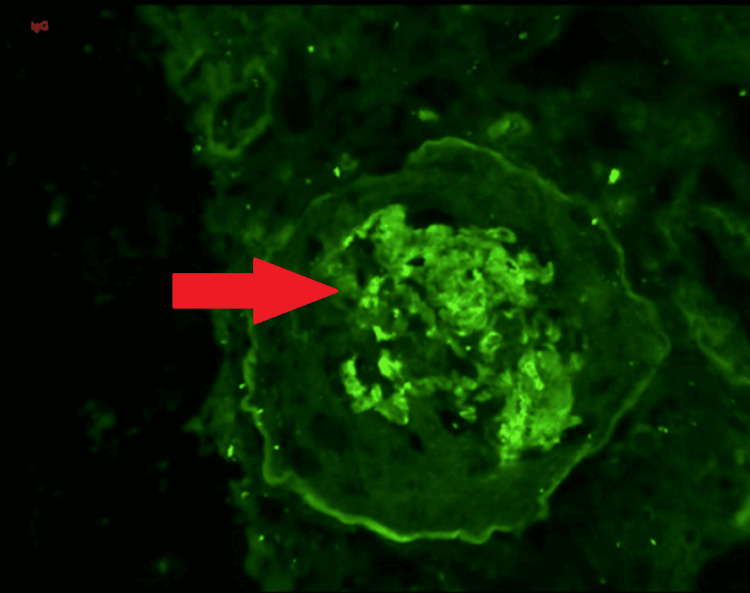
Immunofluorescence (IF) IgG 20X microphotograph showing peripheral linear IgG deposits along the glomerular basement membrane (yellow arrow)

**Figure 3:**
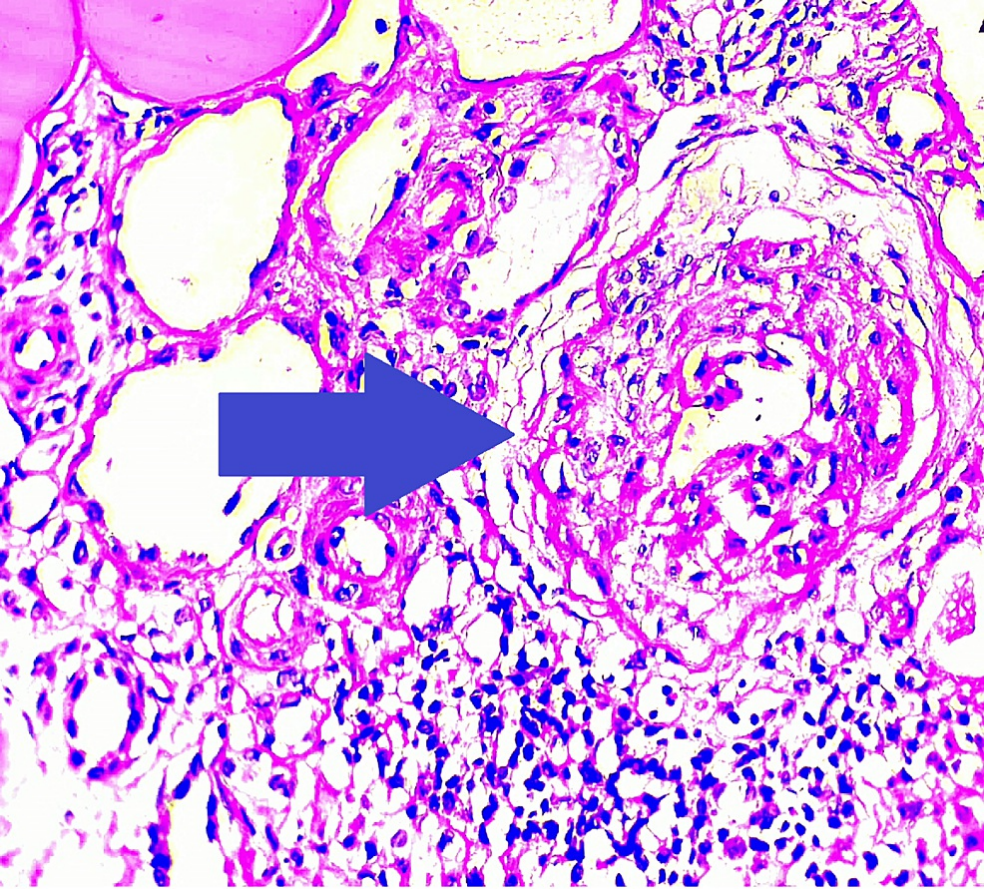
Periodic-acid-Schiff (PAS) stain 40X microphotograph showing a fibrocellular crescent (blue arrow)

Subsequent investigations were conducted, including high-resolution CT (HRCT) of the thorax and bronchoscopy with bronchoalveolar lavage. These tests did not reveal any signs of diffuse alveolar hemorrhage. Figure 1, presented for reference, illustrates predominantly sclerosed glomeruli and one glomerulus with a fibrous crescent on periodic acid-Schiff (PAS) staining. Figure 2 visually represents the histological section through immunofluorescence staining, clearly showing the characteristic linear deposits of IgG along the basement membrane. Figure 3 displays a fibrocellular crescent, providing additional insights into the observed pathological features in this case.

The patient was diagnosed with a sclerosed phase of diffuse crescentic GN attributed to atypical anti-GBM disease. Given the extensive presence of crescents in the kidney biopsy, an aggressive treatment strategy was initiated. This involved administering intravenous methylprednisolone at a dosage of one gram for three consecutive days, followed by oral cyclophosphamide at a daily dose of 100 mg and oral prednisolone, according to the latest guidelines established by the Kidney Disease: Improving Global Outcomes (KDIGO) for anti-GBM disease. While plasmapheresis was recommended as part of the treatment plan, it was ultimately not pursued due to the patient's relatives declining this option. Despite the implementation of these intensive measures, there was no improvement in renal function after 14 days of follow-up, leading to the patient's discharge with the necessity for ongoing maintenance hemodialysis.

## Discussion

Anti-GBM disease is a classic form of small vessel vasculitis mediated by autoantibodies [1]. It tends to occur in two distinct age groups: primarily between 20 and 30 years and again between 60 and 70 years. Notably, it is more prevalent among men in the younger age range [6]. The precise etiology of glomerulosclerosis remains unclear, but certain behaviors and environmental factors may increase susceptibility to the disease [7]. Atypical anti-GBM disease is often triggered by insults to the basement membrane or cross-reactivity with external epitopes induced by various factors such as infections, toxins, neoplasms, renal injury, and endogenous antigens, especially in genetically susceptible individuals. Other factors like exposure to hydrocarbon fumes, metallic dust, tobacco smoke, or substances like cocaine may also contribute [4].

Patients with glomerulosclerosis can present with varying clinical manifestations. Approximately 60 to 80% exhibit both pulmonary and renal symptoms, while 20 to 40% experience renal symptoms exclusively, ranging from mild involvement to rapidly progressive GN with acute renal failure. Less than 10% manifest solely with pulmonary symptoms, which can range from overt pulmonary hemorrhage to inconspicuous bleeding [8].

Detection of GBM antibodies is achieved through direct ELISA, further validated by western blot analysis. Radioimmunoassays or ELISAs for anti-GBM antibodies demonstrate high sensitivity (>95%) and specificity (>97%). Our patient tested negative for anti-GBM antibodies via indirect immunofluorescence (IFA). The absence of seropositivity in atypical anti-GBM disease can be attributed to several factors. Firstly, antibodies against the 4NC1 domain of type IV collagen, as opposed to the 3NC1 domain, may not be detectable by conventional assays. Secondly, these antibodies might lack the capacity to induce inflammation, with IgG4 replacing IgG1, a weaker antibody that cannot activate complement. Finally, the high affinity of antibodies to glomeruli may result in low antibody levels in circulation [9].

Additional laboratory assessments include evaluating levels of anti-neutrophil cytoplasmic antibodies (ANCA), which are positive in 15% to 30% of cases. The preferred diagnostic approach involves a percutaneous kidney biopsy, subsequently examined through light microscopy, immunofluorescence, and electron microscopy. It is theorized that the number of crescents identified in a biopsy correlates with the severity of renal impairment at the presentation time [10].

In treating atypical anti-GBM disease, plasmapheresis may be combined with corticosteroids and cyclophosphamide to optimize therapeutic outcomes. Immunosuppressive medication is essential to prevent the formation of antibodies and the potential rebound hypersynthesis that might occur upon discontinuing plasma exchange. The standard treatment regimen involves high-dose corticosteroids and cyclophosphamide. The five-year survival rate currently exceeds 80%, with fewer than 30% of patients requiring long-term dialysis. In cases of ESRD attributed to glomerulosclerosis, renal transplantation has been utilized as a viable treatment option [11].

## Conclusions

Isolated renal involvement in anti-GBM disease is a rare phenomenon. It should be considered in the diagnostic evaluation of patients presenting with symptoms like pulmonary hemorrhage or anemia. The diagnostic challenge becomes more pronounced when anti-GBM antibodies are absent in the patient's serum. In cases where individuals exhibit clinical features indicative of RPGN, the possibility of atypical anti-GBM disease cannot be dismissed without performing a kidney biopsy. Additionally, a swift clinical deterioration in patients should raise suspicions among attending physicians and nephrologists. In such scenarios, it is crucial to promptly implement a comprehensive management strategy, typically involving a combination of plasmapheresis, cyclophosphamide, and corticosteroids, to address the condition effectively.
